# Antimicrobial stewardship initiative on prescribing at discharge from a community medical center

**DOI:** 10.1017/ash.2025.40

**Published:** 2025-03-14

**Authors:** Gargi Adenkar, Karan Raja, Brandon Chen, Donald Beggs, Christopher Cilderman, Mitesh Patel, Mona Philips

**Affiliations:** 1 Robert Wood Johnson Barnabas Health, Clara Maass Medical Center, Belleville, NJ, USA; 2 Ernest Mario School of Pharmacy, Rutgers, the State University of New Jersey, New Brunswick, NJ, USA

## Abstract

**Objective:**

Assess the impact of a multifaceted discharge antimicrobial stewardship initiative by comparing proportion of appropriate antimicrobial regimens before and after implementation.

**Design:**

Cohort study.

**Setting:**

Non-teaching, urban, community medical center.

**Patients:**

Adult patients prescribed an oral antimicrobial regimen at discharge were included. Patients were randomized irrespective of encounter type or discharge disposition. Pregnant and post-partum patients were excluded.

**Methods:**

A discharge antimicrobial stewardship program was implemented at our facility. Components of the initiative included development of a comprehensive, institution-specific, inpatient and outpatient prescribing guideline, extensive face-to-face clinician education, and real-time, pharmacist prospective audit and feedback at discharge. The validated National Antimicrobial Prescribing Survey tool was used to then categorize one hundred randomized discharge antimicrobial prescriptions as appropriate (optimal or adequate), inappropriate (suboptimal or inadequate), or not assessable. Hospital-specific treatment guidelines, literature references, and patient-specific factors were used to determine appropriateness.

**Results:**

One hundred antimicrobial regimens selected via random sampling were analyzed in each cohort. The proportion of appropriate antimicrobial regimens increased by 15% after program implementation (47% vs 62%, *P* = .03).

**Conclusions:**

Study results highlight the positive impact of a multidisciplinary, multipronged approach in improving discharge antimicrobial prescribing.

## Introduction

According to data from the Centers for Disease Control and Prevention, the United States experiences an annual occurrence of over 2.8 million antimicrobial-resistant infections, and continual emergence of novel resistance mechanisms further exacerbates this issue.^1^ Antimicrobial misuse and associated selective pressure are primary factors perpetuating development and spread of multi-drug resistance. Antimicrobial stewardship programs (ASP) aim to optimize antimicrobial use to preserve their effectiveness, curb resistance, and improve patient outcomes.^2^ ASPs focus a majority of their interventions on hospital inpatients, but efforts to reduce antimicrobial misuse during transitions of care (TOC) and at hospital discharge remain limited. More than one in eight patients receive antimicrobials at hospital discharge and for common infections treated in the inpatient setting, up to 50% of total antibiotic duration occurs post-discharge.^3^ Limited antimicrobial stewardship (AMS) presence makes the TOC setting prone to inaccurate, unnecessary, and prolonged antimicrobial prescribing.^3^

Multiple studies indicate that up to 70% of antibiotic courses prescribed at discharge could be optimized by choosing a safer or more narrow-spectrum antibiotic, reducing duration, or discontinuing antibiotics altogether.^4,5^ Failure to address these issues can increase risk of treatment failure, *Clostridioides difficile* infection, antimicrobial-associated adverse events, and increased healthcare costs.^2^ There is a pressing need for ASPs to allocate more attention and resources to the discharge setting. Research shows that pharmacist-driven AMS interventions incorporating prospective audit and feedback, institution-specific guidelines, and interdisciplinary collaboration can help optimize antibiotic prescribing at discharge.^6–9^ Based on these findings, our facility implemented a comprehensive quality improvement program to enhance discharge antibiotic appropriateness. The study objective was to assess impact of this discharge AMS initiative by comparing appropriateness of discharge antimicrobial regimens before versus after program implementation.

## Methods

This monocentric, pre-post study was approved by the Institutional Review Board and Institutional Review Committee with receipt of an informed consent waiver. The study was conducted at a 472-bed, urban, acute care, non-teaching, community medical center part of a larger health system. The hospital’s AMS Committee consists of 40 representatives from medicine, pharmacy, nursing, administration, quality, and infection control. The Committee conducts pre-authorization and prospective audit and feedback interventions impacting the majority of inpatients, specifically concentrating on antimicrobial selection and dosing, de-escalating broad-spectrum antimicrobials, optimizing total duration of therapy, conducting intravenous-to-oral conversions, and identifying candidates for beta-lactam allergy assessment and skin testing. In addition to participating in multidisciplinary infectious diseases (ID) rounds, the ID pharmacist serves as a primary resource for provider consultation. While the impact of some stewardship interventions permeate to discharge prescriptions, there remains significant opportunity for augmenting TOC stewardship guidance and resources.

To improve prescribing practices at discharge, the AMS Committee implemented a multifaceted discharge AMS program that consisted of three primary components: (1) development and dissemination of a comprehensive, institution-specific emergency department (ED), inpatient, and outpatient antimicrobial prescribing guidance document, (2) provision of hospital-wide ASP-based clinician education, and (3) real-time, direct, pharmacist prospective audit and feedback on oral antimicrobials electronically prescribed at discharge.

The institution-specific prescribing guideline was developed to standardize antimicrobial prescribing across different hospital service lines and professional disciplines. The guideline was developed by and underwent review and approval from the AMS Committee, as well as various services including ID providers, ED providers, Intensivists, Hospitalists, advanced practice providers (APP), and the case management teams. The guideline includes first-line empiric antimicrobial recommendations for twenty common infectious conditions encountered in ED, inpatient, and outpatient settings. Furthermore, it provides alternative antimicrobial choices, literature-supported therapy durations, oral step-down therapy guidance, management strategies for patients with varying severities of beta-lactam allergies, and de-escalation approaches based on clinical improvement and pertinent laboratory parameters. These recommendations were formulated based on primary literature, pathogen isolation frequencies, and local susceptibility rates as outlined in the local and state antibiogram. Physical copies were distributed to the ED, Intensive Care Unit (ICU), and the on-site retail pharmacy, while electronic copies were disseminated to providers via email.

Numerous educational presentations on discharge antimicrobial prescribing pearls were provided to clinicians, including the ED physicians and APPs, inpatient pharmacists, and on-site retail pharmacists. These included in-services, committee presentations, and grand rounds. Additionally, an educational assignment on the hospital’s digital learning platform was deployed to all employees to reinforce previously delivered education.

The last component of the initiative involved direct pharmacist evaluation and intervention on discharge antimicrobial orders. The pharmacist workflow consisted of three main steps: screening, assessment, and intervention. During screening, the pharmacist reviewed an electronic report twice daily to identify patients pending discharge or who had been discharged within the past 24 hours and were prescribed an oral antimicrobial regimen electronically. Only oral antimicrobial regimens were subject to review and assessment. Using the institution-specific prescribing document, the pharmacist evaluated regimen appropriateness and, if necessary, provided recommendations to optimize therapy to the prescribing team. If the recommendation was accepted, the pharmacist documented the intervention and requested the prescriber to send an updated prescription to the patient’s pharmacy. The pharmacist directly coordinated with the outpatient pharmacy to ensure the older prescription was canceled and the patient would collect the updated filled prescription. Education on this standardized workflow was provided to the pharmacist investigators with emphasis placed on optimizing efficiency while balancing other responsibilities.

Our standardized workflow and antimicrobial prescribing guideline aimed to minimize both subjectivity of pharmacist assessment and need for consultation with an ID physician or pharmacy specialist. Clear and measurable definitions were used to determine antimicrobial regimen appropriateness. For instance, our study utilized a quantifiable and objective definition of inappropriate duration of antimicrobials with consideration of missed doses or nonstandard administration times. Even within our antimicrobial prescribing guideline, recommendations for use of first-line antimicrobials accounted for local susceptibility data using our hospital and regional antibiograms and assigned cutoffs for first-line versus alternative antimicrobial recommendations. Cutoffs were determined through epidemiological and antimicrobial susceptibility testing studies and specific guideline recommendations.

Stewardship interventions often required close coordination with both inpatient and retail pharmacy teams. The on-site retail pharmacy was instrumental in identifying AMS opportunities and escalating potential discrepancies or directly intervening prior to dispensing. Pharmacists rounding with the medical team identified patients eligible for discharge antimicrobial intervention and coordinated with the retail pharmacist. Intervening pharmacists actively communicated with all outpatient pharmacies to ensure seamless and equitable TOC while widening our reach. Pharmacists documented interventions on three platforms: an online clinical surveillance platform, an intervention tracker in Excel, and within the electronic health record (EHR) pharmacist intervention section.

The antimicrobial prescribing protocol was finalized and disseminated in January 2023, and educational initiatives were launched at that time. The intervention period began in January 2023 and continued throughout the data collection period, from February 1^st^ to March 1^st^, 2023. While most educational sessions occurred in January and February 2023, we extended implementation of the stewardship initiative beyond the post-intervention period. These post-intervention efforts included further dissemination of the prescribing guideline to Hospitalists, APPs, and any groups that either did not receive education during the initial phase or requested re-education. Re-education was emphasized to ensure broad and continued protocol engagement across all relevant groups.

All adult patients who received electronically prescribed oral antimicrobials at discharge were evaluated for appropriateness and intervention, regardless of encounter type or discharge disposition. Patients were excluded if they were pregnant or post-partum or discharged on parenteral antimicrobials. Pregnancy status was confirmed through EHR review with pregnancy lab testing. The pre- and post-intervention cohorts consisted of patients discharged between January 1–31^st^, 2022 and February 1^st^ to March 1^st^ 2023, respectively. These dates were selected to limit potential impact of seasonality on prescribing.

Antimicrobial appropriateness was determined using institution-specific prescribing guidelines, relevant literature references, and patient-specific factors. The validated National Antimicrobial Prescribing Survey (NAPS) tool was utilized to evaluate compliance of antimicrobial regimen components (antimicrobial choice, dosing, frequency, duration, and allergy mismatch) to guideline recommendations. Based on the composite of individual components, the NAPS tool categorizes appropriateness into five defined levels: optimal, adequate, suboptimal, inadequate, or not assessable.^10^ Optimal or adequate regimens were classified as appropriate and suboptimal or inadequate regimens as inappropriate.

EHR reviews were conducted to gather relevant information including prescriber notes and discharge diagnoses to determine antimicrobial selection appropriateness for the specified indication. Patient-specific factors, such as age, height, weight, serum creatinine, liver function tests, and microbiology data determined appropriateness of dosing and frequency. Total therapy duration was calculated by adding inpatient and outpatient days of antimicrobial therapy. Inpatient days of therapy were counted if patients received at least 75% of the total scheduled antimicrobial doses for the day. Excessive duration was defined as a duration exceeding 2 days beyond the recommended period. The same two pharmacist investigators independently evaluated regimen appropriateness pre- and post-intervention. In case of any discrepancies in NAPS tool assessments between pharmacists, a third pharmacist conducted a final review. All pharmacists involved were trained in AMS/ID practices and engaged in stewardship activities within their respective roles.

The primary outcome was the proportion of appropriate discharge antimicrobial prescriptions before and after stewardship initiative intervention. Exploratory outcomes included proportion of each NAPS category, prescribing trends based on provider type, intervention type and acceptance rates, and appropriateness of antimicrobial choice, dose, frequency, duration, and allergy mismatch.

A sample size of 100 antimicrobial regimens in each cohort would provide a 90% statistical power at a two-sided alpha of 0.05 to detect an anticipated 20% increase in antimicrobial appropriateness after program implementation based on data from previous studies.^6,8,11–13^ Therefore, 100 antimicrobial regimens were selected for each cohort through simple random sampling to represent the population in the corresponding study period. Although 100 regimens were chosen as the study sample, pharmacists attempted to intervene on all regimens in the post-intervention period. Categorical data were assessed with the Chi-square test. Descriptive statistics were used to evaluate exploratory outcomes.

## Results

**Figure 1.**
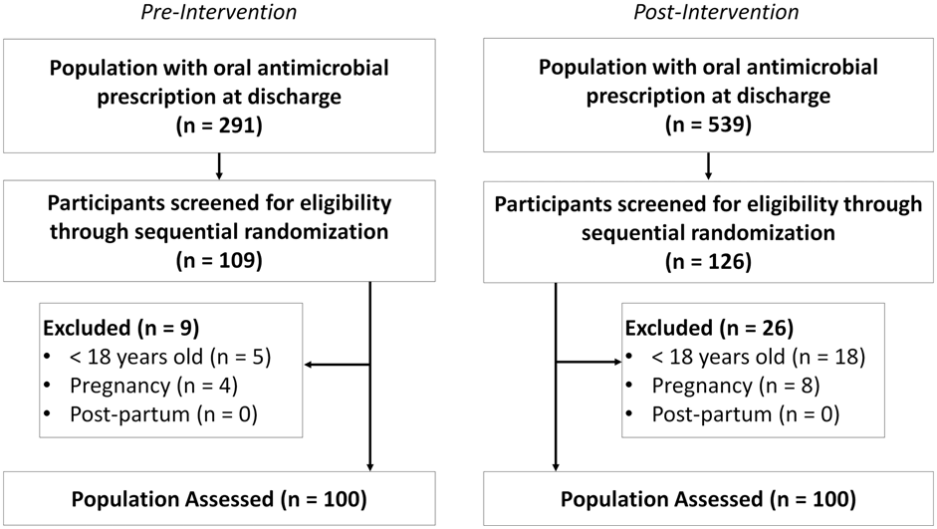
Enrollment.

In January 2022, 291 adult patients were discharged with an oral antimicrobial regimen prior to stewardship initiative implementation. Among these patients, 109 participants were screened for eligibility through simple random sampling. After excluding 9 ineligible participants, 100 participants were included in the pre-intervention cohort. In February 2023, 539 adult patients were discharged with an oral antimicrobial regimen. Through simple random sampling of 126 participants, 100 were included in the post-intervention cohort after excluding 26 (see Figure 1).

No significant differences were observed between the two groups in baseline demographics, clinical characteristics, and antimicrobial indications (refer to Table 1). The mean age of patients in the pre- and post-intervention groups was 46 and 45 years, respectively (*P* = .66). The proportion of female patients was slightly higher in the post-intervention cohort compared to the pre-intervention cohort (69% vs 59%, *P* = .14). The majority of patients in both the pre- and post-intervention cohorts were directly discharged from the ED (75% vs 71%, *P* = .52). Most antimicrobial regimens were prescribed by ED APPs (56% vs 67%), followed by ED physicians (19% vs 4%) and inpatient practitioners (25% vs 29%) in the pre- versus post-intervention cohorts, respectively.

**Table 1.** Baseline characteristics

Characteristic	Pre-intervention	Post-intervention	*P*-value
Age – year, mean (± SD)	46 (19.8)	45 (18.7)	0.66
Female sex – no. (%)	59 (59)	69 (69)	0.14
Inpatient admission – no. (%)	25 (25)	29 (29)	0.52
Immunocompromised^a^ – no. (%)	13 (13)	6 (6)	0.09
Complete antibiotic allergy history (drug and reaction) – no. (%)	9 (75)	7 (77.8)	1
Antimicrobial indication – no. (%)			
Urinary tract	26 (26)	26 (26)	1
Pulmonary	15 (15)	11 (11)	0.29
Skin and soft tissue	22 (22)	22 (22)	1
Intra-abdominal	22 (22)	14 (14)	0.14
Other^b^	15 (15)	27 (27)	0.04

a Immunocompromised was defined as having one or more of the following: chronic renal failure (stage 4 or 5), nephrotic syndrome, hemodialysis or peritoneal dialysis, congenital or acquired asplenia, congenital or acquired immunodeficiency, generalized malignancy, HIV (untreated or advanced: CD4 count of 200 or less), hematological malignancies with active treatment, sickle cell disease/thalassemia, solid organ or bone marrow transplant within 2 years, high-dose long-term steroids (20 or more mg of prednisone or equivalent per day when administered for 2 or more weeks), neutropenic (absolute neutrophil count of ≤ 500 cells/ml), or on immunosuppressive medications (alkylating agents, antimetabolites, transplant-related immunosuppressive drugs, tumor necrosis factor blockers, immunosuppressive or immunomodulatory agents used in auto-immune conditions).

b The infection types that fell under the “Other” category are as follows: ENT (acute otitis media, dental infection, tonsillitis, acute pharyngitis, and sinusitis), diabetic foot infection, pelvic inflammation disease (cervicitis, vaginitis, and urethritis), sexually transmitted diseases, bacterial vaginosis, and shingles.

**Figure 2.**
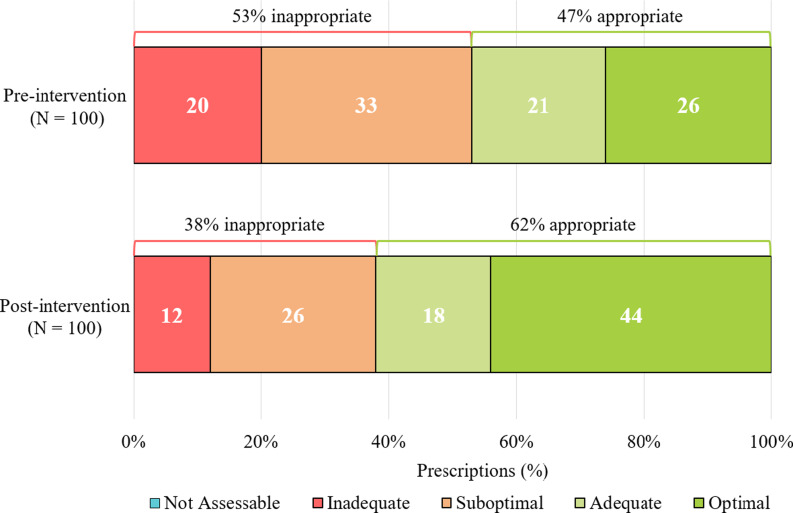
Proportion of each NAPS category and overall appropriateness of antimicrobial prescriptions.

**Figure 3.**
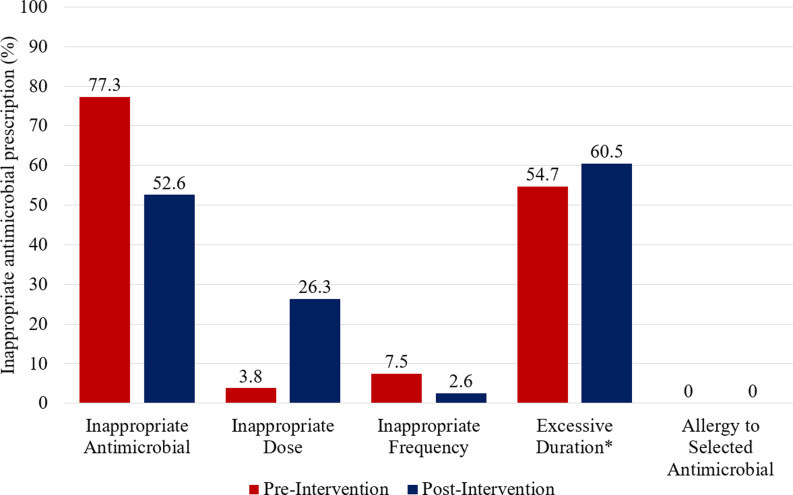
Reasons antimicrobial regimens considered inappropriate. *Note*: Regimens may have had more than one reason for being inappropriate. *Defined as > 2 days from recommended duration.

Overall, the proportion of appropriate antimicrobial regimens increased by 15% following initiative implementation (47% vs 62%, *P* = .03). Exploratory outcome analyses indicated an increase in optimal regimens (26% vs 44%) and a decrease in suboptimal (33% vs 26%) and inadequate (20% vs 12%) therapy (see Figure 2). Of the 100 patients in the post-intervention group, 68 received direct pharmacist intervention. Detailed data can be found in Figure 3 and Table 2.

**Table 2.** Pharmacist interventions and outcomes

Pharmacist interventions and outcomes	Prescriptions assessed, n (%) [n = 68]
No intervention required	42 (61.8)
Intervention made and accepted	9 (13.2)
Broad-spectrum	2 (22.2)
Excessive duration	1 (11.1)
Inadequate regimen	1 (11.1)
Inappropriate dosing	3 (33.3)
Multiple reasons	2 (22.2)
Intervention made and not accepted	3 (4.4)
Inadequate regimen	1 (33.3)
Excessive duration	2 (66.7)
Intervention not made	10 (14.7)
Broad-spectrum	4 (40)
Excessive duration	2 (20)
Inadequate regimen	1 (10)
Multiple reasons	3 (30)
Unable to reach prescriber	4 (5.9)
Broad-spectrum	1 (25)
Excessive duration	2 (50)
Inadequate regimen	1 (25)

## Discussion

Implementation of a discharge AMS initiative significantly increased the proportion of appropriate electronically prescribed oral antimicrobial regimens. Our study evaluated the composite of antimicrobial regimen appropriateness in contrast to previously published studies which focused on individual constituents as their primary endpoint. Literature evaluating the impact of stewardship at discharge is limited. Our study contributes to the existing body of research by employing a validated tool to assess appropriateness, implementing interventions through a handshake stewardship approach, employing a thorough but replicable pharmacist workflow, and including a wide range of infection types treated with oral antimicrobials.

Our study evaluated appropriateness utilizing the NAPS tool. This tool is recognized and validated as a standardized assessment used to quantify and qualify antimicrobial appropriateness. It has been utilized for accreditation purposes and to strengthen stewardship strategies.^14^ NAPS is specific to antimicrobials and provides a simplified and less labor-intensive approach to differentiating and identifying areas for improvement within a regimen compared to other tools used in similar studies, such as the validated Pharmaceutical Care Network Europe Drug-Related Problem classification tool.^15^ Another notable element of the NAPS tool is inclusion of the “not assessable” category. When evidence was insufficient to assess appropriateness, pharmacists could mark the regimen as “not assessable” rather than introducing subjectivity and skewing accuracy of study results. This facet differs from other studies that assumed appropriateness when lacking evidence.

To our knowledge, only one previous study utilized the NAPS tool to evaluate antimicrobial regimen appropriateness. This retrospective study found that 76% of prescribed oral antibiotics were deemed appropriate overall for patients discharged from the ED.^5^ This study also analyzed prescribing trends based on provider type and found that residents had the highest percentage of appropriate prescriptions, followed by attending physicians and APPs. Contrarily, our institution is a non-teaching facility and does not have frequent turnover of medical residents or fellows, supporting sustainability of stewardship efforts. The relatively stable provider composition during the pre- and post-intervention periods likely limits potential for significant bias due to differences in provider training. Although our study collected data on provider type, no associated prescribing trends were identified and the majority of patients analyzed were directly discharged from the ED. For this reason, our educational efforts and pharmacist interventions were targeted toward ED physicians and APPs.

An interesting observation is the significant increase in patients discharged on oral antimicrobials in February 2023 compared to January 2022. This trend may signal that the hospital-wide educational initiatives and increased AMS emphasis contributed to prescriber behavior change, indicates greater comfort with utilizing oral antimicrobials, and reflects a broader cultural shift toward more appropriate oral antimicrobial use.

Parsels et al. evaluated appropriateness of discharge oral antimicrobial prescriptions after AMS efforts.^15^ Their ASP includes an adult ID pharmacist, a pediatric ID pharmacist, a faculty ID pharmacist, and a post-graduate year 2 (PGY-2) ID pharmacy resident. Interventions and antimicrobial reviews were conducted by these ID pharmacists who consistently round with ID medical teams and follow patients through discharge. While our AMS team includes an adult ID pharmacist, non-ID-trained clinical pharmacists were involved in program implementation and daily workflow. We did not specifically measure pharmacists’ time to review and intervene on antimicrobial regimens during the post-intervention period, but anecdotally we estimate the workflow required a review time of 10 minutes per patient. As such, our study methods may be used as a blueprint for hospitals with limited AMS resources or non-ID-trained pharmacists to practice AMS in the discharge setting.

Interestingly, the observed absolute difference of 15% between the pre- and post-intervention cohorts was comparatively lower than findings of a previous study with similar endpoints.^6^ Following the intervention, the study reported an increase in optimal antimicrobial prescriptions from 36% to 81.5% (*P* < .001), along with a reduction in severe antimicrobial-related adverse effects (3.2% vs 9%) and a decrease in total antimicrobial duration (time-adjusted absolute difference, −1.1 [95% CI, −1.7 to −0.6] antibiotic days) in the post-intervention group. However, in our facility, previous interventions and discussions surrounding discharge antimicrobial prescriptions had already increased appropriateness to a higher baseline before study implementation. This may account for the relatively smaller difference observed in our primary outcome compared with previous studies.

Intervention timing likely impacted study results. Conducting educational initiatives closer to the post-intervention period likely enhanced retention and facilitated more immediate clinical practice application and changed prescribing behavior. Additionally, we provided frequent re-education to reinforce key concepts.

We developed targeted education based on data analyzed during the pre-intervention period. There were 2 main reasons antimicrobial regimens were considered inappropriate: antimicrobial selection and excessive duration. Our educational presentations focused on optimizing antimicrobial selection by comparing commonly used antimicrobials with limited efficacy due to resistance at our institution with alternative options with preserved efficacy. We also emphasized reducing total duration of therapy by providing updated clinical trial data on non-inferiority of shorter versus longer antibiotic durations and presented de-escalation techniques. Although we successfully reduced the percent of regimens with inappropriate antimicrobial selection in our post-intervention cohort, the percent of regimens with excessive duration and inappropriate dosing numerically increased. The regimens in our post-intervention period with inappropriate dosing used dosing not recommended in our prescribing guideline but may have been recommended in a newly available tertiary reference at our hospital during the study period. A greater proportion of patients within the post-intervention group with excessive durations also had inpatient admission, with these regimens continuing at discharge. In contrast, patients in the pre-intervention group with excessive durations had no inpatient admission, which may explain the observed increase. However, this should be considered hypothesis-generating as statistical analyses were not performed on these data, precluding definitive conclusion.

Several limitations should be considered. Firstly, the NAPS tool has limited assessment of appropriateness based on antimicrobial safety, which plays a significant role in determining optimal therapy for pregnant and pediatric patients. Due to the lack of sufficient validation studies in these populations, we excluded pediatric and pregnant patients to mitigate potential limitations. Secondly, variability in interpretation of the NAPS tool among independent investigators may introduce subjectivity, as reflected in concordance rates of 63% and 64% for the pre- and post-intervention group assessments, respectively. This variability could have influenced results and highlights the need for consistent application of the tool. Another limitation, similar to other comparator studies, stemmed from the assumption that antibiotics were indeed indicated for the infections they were intended to treat.^15,16^ This assumption was made as pharmacists assessing regimen appropriateness did not follow antimicrobial use throughout the course of therapy, but only on day of discharge. As a result, our study relied solely on prescriber discretion to determine need for antimicrobial treatment. However, discussion between the AMS and treating team was common practice if antimicrobial need was not concrete. Our study also exclusively evaluated patients prescribed oral antimicrobials at discharge, excluding those appropriately not prescribed antimicrobials. This is particularly relevant in the ED, where asymptomatic, positive urine cultures, for instance, often do not warrant antimicrobials. By not capturing these cases, our analysis may underestimate the stewardship initiative’s broader impact.

Real-time data availability, limited AMS EHR integration, and reliance on physical guidelines restricted study reach. Physical guidelines lack the adaptability and accessibility of electronic versions, which allow for real-time updates and broader dissemination. We elected to initially distribute physical copies because electronic distribution required several administrative steps and an extended approval process, delaying availability at time of implementation. Furthermore, both ED and ICU providers in our facility frequently relied on physical references, paralleling current practice. In the future, guideline accessibility via institutional intranet, information technology involvement, and clinical decision support systems will be leveraged to optimize stewardship impact and reach.

Beyond these methodological and logistical challenges, certain study design biases could affect reliability of findings. The possibility of the Hawthorne effect may have inflated the intervention’s estimated effect, as prescribers aware of being observed might have altered behavior to align with guidelines. Maturation bias resulting from education and exposure over time might have naturally improved prescribing between the pre- and post-intervention periods, making it difficult to isolate the intervention’s impact. Lastly, there remains a possibility of regression to the mean, as observed improvements in prescribing behavior may diminish over time following study completion without sustained reinforcement of stewardship efforts. Together, these factors underscore the importance of ongoing stewardship education and evaluation to ensure enduring improvements.

## Supporting information

Adenkar et al. supplementary material Adenkar et al. supplementary material

## Supplementary material

For supplementary material accompanying this paper visit http://dx.doi.org/10.1017/ash.2025.40. click here to view supplementary material
