# Supplementary material for: Antimicrobial stewardship initiative on prescribing at discharge from a community medical center
Source: Antimicrob Steward Healthc Epidemiol. 2025 Mar 14;5(1):e76. doi: 10.1017/ash.2025.40 (PMC11920916; doi:10.1017/ash.2025.40)
Supplement: Adenkar et al. supplementary material [file S2732494X25000403sup001.pptx]

## Slide 1
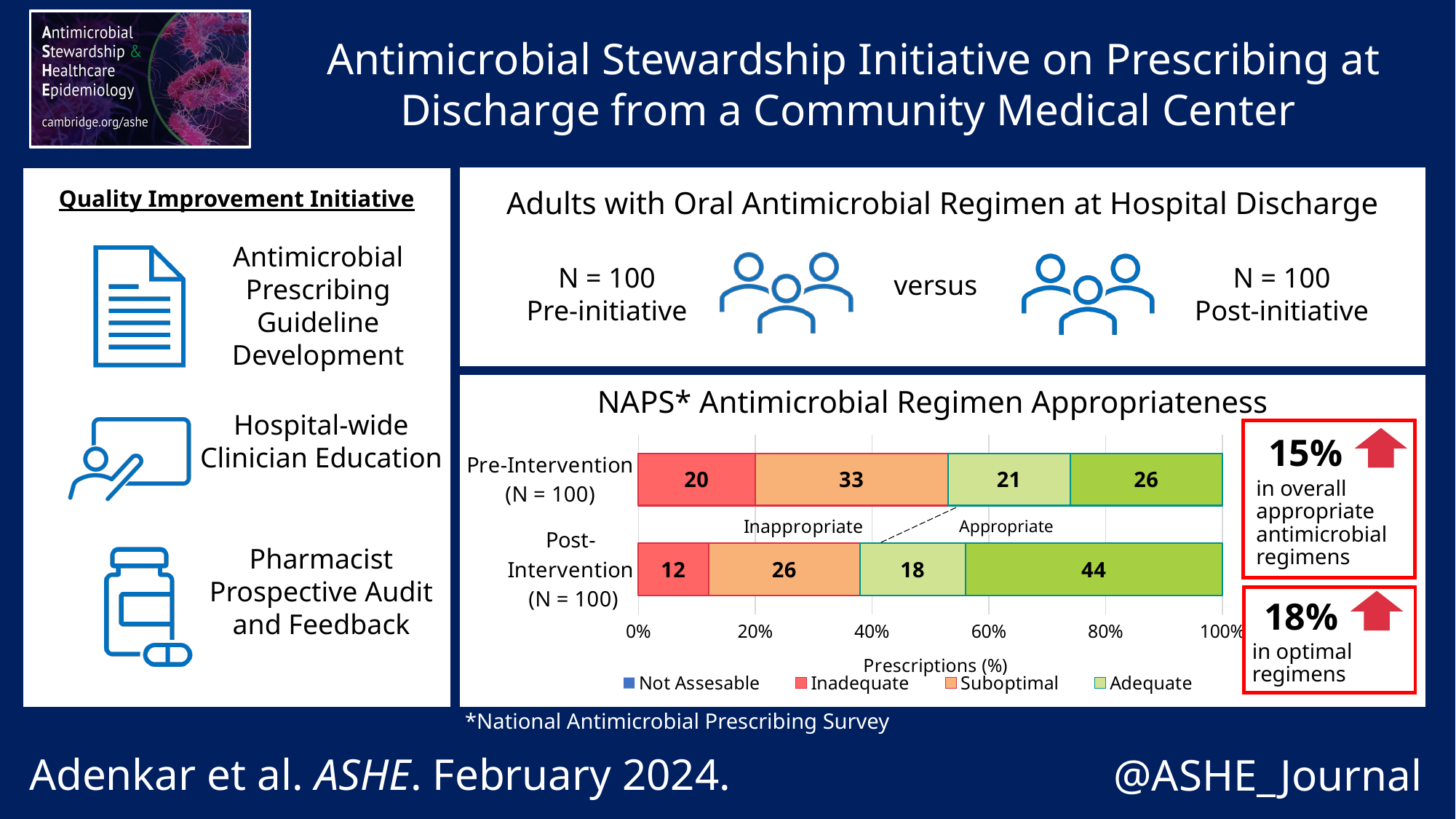

Antimicrobial Stewardship Initiative on Prescribing at Discharge from a Community Medical Center
3.5
Adults with Oral Antimicrobial Regimen at Hospital Discharge
Quality Improvement Initiative
Antimicrobial Prescribing Guideline Development
N = 100
Pre-initiative
N = 100
Post-initiative
versus
NAPS* Antimicrobial Regimen Appropriateness
### Chart
| Category | Not Assesable | Inadequate | Suboptimal | Adequate | Optimal |
|---|---|---|---|---|---|
| Post-Intervention
 (N = 100) | 0.0 | 12.0 | 26.0 | 18.0 | 44.0 |
| Pre-Intervention
(N = 100) | 0.0 | 20.0 | 33.0 | 21.0 | 26.0 |Hospital-wide Clinician Education
15%
in overall appropriate antimicrobial regimens
18%
in optimal regimens
Appropriate
Pharmacist Prospective Audit and Feedback
*National Antimicrobial Prescribing Survey
Adenkar et al. ASHE. February 2024.
@ASHE_Journal
